# Glomerulopathy

**DOI:** 10.1093/emph/eoab015

**Published:** 2021-05-25

**Authors:** Noel T Boaz, Robert L Chevalier

**Affiliations:** 1 Laboratory of Biological Anthropology and Anatomy, Integrative Centers for Science and Medicine, Martinsville, VA 24112, USA; 2 Department of Pediatrics, University of Virginia School of Medicine, Charlottesville, VA 22901, USA

## DESCRIPTION OF THE CONDITION

Glomerulopathy is generic disease of the renal glomerulus, impairment of which can lead to hematuria or proteinuria due to injury or dysfunction of the endothelium, glomerular filtration barrier or podocyte [1]. The Nephrotic Syndrome (proteinuria, hypoalbuminemia, edema and hyperlipidemia) develops in the context of heavy proteinuria, and the extra-renal components (edema and hyperlipidemia) have more elusive etiologies. A renal ontophylogenetic approach following Homer Smith [2] assists in establishing a firm empirical basis for further clinical investigation.

## EVOLUTIONARY PERSPECTIVES

The most primitive ki dney was the pronephros, evolved as a urea-secreting organ in a multicellular animal [2] that was isotonic with its marine environment [3] some 950 million years ago (Ma) [4]. The glomerulus evolved in the mesonephric kidney of early chordate ancestors (684–824 Ma), with its primary function being the removal of excess water flooding into the body from its freshwater environment [2–5]. The metanephric kidney was a terrestrial vertebrate adaptation (350 Ma) conserving metabolic water, thus essentially reversing the earlier mesonephric adaptation [2–5]. The modern human kidney retains embryonic derivatives and remanent functions of these renal primordia. Edema resulting from glomerulopathy can be due to either reduced oncotic pressure or to increased sodium retention in the aldosterone-sensitive distal nephron [5] activated by increased plasminogen leaked by a damaged filtration barrier [7], a water-saving adaptation inherited from our terrestrial vertebrate ancestors.

## FUTURE IMPLICATIONS

Diagnosing pathology and assessing etiology on the basis of dysfunction of evolutionary adaptations is a promising approach to analyzing contemporary disease and injury [8]. An evo-devo perspective has proved effective in explicating the complex etiology of edema in the nephrotic phenotype [3, 6, 7] in glomerular disease. The etiology of hyperlipidemia resulting from glomerulopathy remains unclearly explained and is a challenge for the future.
